# Photoactive Yellow Protein Adsorption at Hydrated Polyethyleneimine and Poly-l-Glutamic Acid Interfaces

**DOI:** 10.3390/molecules28104077

**Published:** 2023-05-13

**Authors:** Szilvia Krekic, Mark Mero, Michel Kuhl, Kannan Balasubramanian, András Dér, Zsuzsanna Heiner

**Affiliations:** 1 School of Analytical Sciences Adlershof, Humboldt-Universität zu Berlin, 12489 Berlin, Germany; 2 Institute of Biophysics, Biological Research Centre, 6726 Szeged, Hungary; 3 Doctoral School of Multidisciplinary Medical Sciences, University of Szeged, 6720 Szeged, Hungary; 4 Max Born Institute for Nonlinear Optics and Short Pulse Spectroscopy, 12489 Berlin, Germany; 5 Department of Chemistry and IRIS Adlershof, Humboldt-Universität zu Berlin, 12489 Berlin, Germany

**Keywords:** polyelectrolyte, vibrational sum frequency generation spectroscopy, photoactive yellow protein, polyethyleneimine, poly-l-glutamic acid, layer-by-layer deposition

## Abstract

Chiral and achiral vibrational sum-frequency generation (VSFG) spectroscopy was performed in the 1400–1700 and 2800–3800 cm^−1^ range to study the interfacial structure of photoactive yellow protein (PYP) adsorbed on polyethyleneimine (PEI) and poly-l-glutamic acid (PGA) surfaces. Nanometer-thick polyelectrolyte layers served as the substrate for PYP adsorption, with 6.5-pair layers providing the most homogeneous surfaces. When the topmost material was PGA, it acquired a random coil structure with a small number of β_2_-fibrils. Upon adsorption on oppositely charged surfaces, PYP yielded similar achiral spectra. However, the VSFG signal intensity increased for PGA surfaces with a concomitant redshift of the chiral C^α^-H and N–H stretching bands, suggesting increased adsorption for PGA compared to PEI. At low wavenumbers, both the backbone and the side chains of PYP induced drastic changes to all measured chiral and achiral VSFG spectra. Decreasing ambient humidity led to the loss of tertiary structure with a re-orientation of α-helixes, evidenced by a strongly blue-shifted chiral amide I band of the β-sheet structure with a shoulder at 1654 cm^−1^. Our observations indicate that chiral VSFG spectroscopy is not only capable of determining the main type of secondary structure of PYP, i.e., β-scaffold, but is also sensitive to tertiary protein structure.

## 1. Introduction

The interior of cells is far from being a pure electrolyte, as it contains a lot of solvated macromolecules and cytoskeletal components [1,2]. Under such circumstances, biochemical reactions are dominated by macromolecular interactions taking place at charged interfaces between proteins or nucleic acids and supramolecular assemblies (composed of protein biopolymers, planar or cubic lipid phases, etc.), inside a dynamic, aqueous environment [3,4,5,6,7]. It was shown that the dynamics of a model peptide at the interface significantly deviates from that observed in the bulk [8], implying that proper characterization of the behavior of peptides and proteins at macromolecular interfaces, including their reversible adsorption to surfaces, is a prerequisite for understanding fundamental physiological processes stemming from intracellular reactions [9]. Upon adsorption, proteins self-assemble into viscoelastic 2D nanolayers [10,11], depending on the overall conditions at the interfaces, such as material surface parameters, surface charge, pH, concentration of proteins, etc. [12,13,14,15]. However, the adsorption of a protein is also highly influenced by its amino acid sequence and its higher-order structure.

A detailed knowledge about these complex phenomena, dominated by electrostatic, van der Waals, and hydrophobic interactions, is also crucial when designing biomaterials for implants, medical devices, and bioelectronics. On the other hand, photosensitive coatings based on biomolecules are attractive due to their biodegradability and easy manufacturability for the conversion of light to electrical energy [16], and for integrated optics [17,18,19]. In such applications, a controlled immobilization of proteins is needed to create well-ordered protein layers with high optical quality.

All-in-all, understanding and controlling macromolecular interactions with biocompatible surfaces at the nanoscale, in their nearly natural, aqueous environment, is necessary from the point of view of both basic biochemistry and the development of advanced functional biomaterials and biosensors. In situ determination of the orientation and secondary structure of the adsorbed biomolecules at interfaces would be required, but the atomic-level description of the interacting macromolecular interfaces is also still to come.

To this end, methods of measurement based on physical principles (e.g., infrared and Raman techniques, CD spectroscopy, neutron scattering, micro-calorimetry, electron microscopy, and evanescent-wave-based techniques, such as OWLS and interferometry) are widely used [20,21,22,23,24,25,26], but combined chemical and surface specificity is usually missing. Vibrational sum-frequency generation (VSFG) spectroscopy is a powerful, label-free technique, which is especially suited for such investigations, as it is sensitive only to anisotropic molecular structures that are characteristic to the immediate vicinity of macromolecular interfaces, and, at the same time, it retains the chemical sensitivity of infrared spectroscopy [27,28,29,30,31]. In addition to surface specificity and structural information, the VSFG technique is also capable of revealing molecular chirality [32,33,34] and secondary structure of proteins [30,35,36], in a very similar manner to the methods based on vibrational circular dichroism (VCD) [37,38] and Raman optical activity (ROA) [39,40,41].

Photoactive yellow protein (PYP) is a molecule with a high potential for photosensitive coating applications. PYP is a water-soluble light-sensing protein from the microorganism *Halorhodospira halophila*. PYP is a hydrophilic, 14 kDa protein consisting of 125 amino acids and its structure often serves as a model for understanding biologically important photoreceptors, such as rhodopsins, and receptor activation in biological signal transduction processes [42,43,44]. Recently, PYP received more attention due to its possible application in bioelectronics and integrated optics [18,19,45,46,47]. PYP belongs to the group of globular proteins and is the model structure for the PAS-(Per–Arnt–Sim) domain superfamily, which is a signal transduction α/β pathway structure commonly found in prokaryotic and eukaryotic organisms [48]. PAS domains are key components in sensory and signaling proteins, and they are an integral part of the protein–protein interactions taking place during signaling. The α/β pathway structure of PYP consists of a central β-sheet with five strands and helical connectors on both sides. On the sides of the β-sheet, the PYP molecule has two hydrophobic cores [49,50,51]. The molecular surface of PYP includes several patches which have both negative and positive electrostatic potential since mostly polar amino acids can be found on the outer side that interact with solvents via dipole–dipole interactions. Being a highly soluble protein, PYP has all its important hydrophilic parts outside, forming a water shell. Therefore, hydrogen bonds as driving forces play a determinative role besides ionic interactions. In contrast, the apolar amino acids sit in the interior part of the protein but are also expected to play a role in conformational changes assumed to accompany the adsorption process [52].

In this paper, we report on a model study carried out by using high-spectral-resolution, 100-kHz VSFG spectroscopy [53,54] on photoactive yellow protein and its adsorption on self-assembled layers of biopolymers of different electric charge. The macromolecular surface interacting with PYP was built up from polyethyleneimine (PEI) and poly-L-glutamate (PGA) chains via layer-by-layer (LbL) deposition, forming positively and negatively charged layers on the surface of a CaF_2_ substrate, respectively [55]. Here, we extensively characterize the conformational changes taking place upon the interaction of our model protein, PYP, with positively charged PEI and negatively charged PGA interfaces at the molecular level, by applying a self-developed VSFG setup. Chiral- and achiral-mode spectra also reveal orientational information of both the adsorbed protein and the water molecules at the interface. The results enable deeper insight into protein interaction processes at biological interfaces, and their generalizable implications for basic and applied sciences are briefly discussed.

## 2. Results and Discussion

### 2.1. VSFG Spectra of Hydrated PYP Films

**Figure 1:**
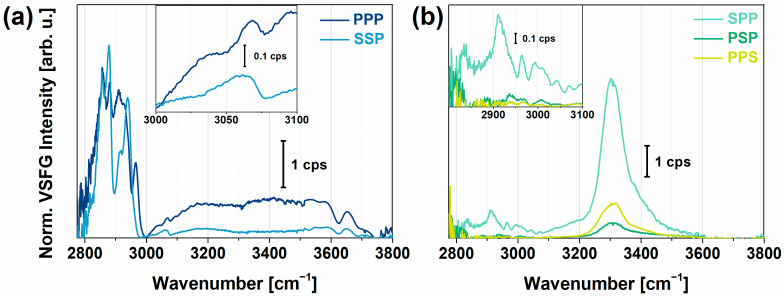
(**a**) Achiral (SSP, PPP) and (**b**) chiral (SPP, PSP, PPS) VSFG spectra of a hydrated thin film of PYP on CaF_2_. The spectra were interlinked at 3500 cm^−1^ from two measurement sets between 2800–3500 and 3000–3800 cm^−1^. The insets show the zoomed-in view in the 3000–3100 cm^−1^ (**a**) and the 2800–3100 cm^−1^ range (**b**).

To determine the VSFG spectrum of PYP without the effects of the charged polyelectrolyte layers, PYP films were created on top of plasma-treated CaF_2_ slabs. These slabs were kept at constant humidity and temperature during the experiments. VSFG spectra measured for a high-humidity (RH > 85%) environment are shown in Figure 1. The spectra of the hydrated thin protein films were acquired in different chiral and achiral polarization combinations with the spectral range of the broadband mid-infrared (MIR) laser beam tuned to either 2800–3500 cm^−1^ or 3000–3800 cm^−1^, to cover the C–H, N–H, and O–H stretching regions. The VSFG spectra recorded in the two cases were joined at 3500 cm^−1^ (Figure 1).

In achiral polarization combinations (PPP and SSP), intense C–H stretching bands were visible between 2800 and 3000 cm^−1^ that can be assigned to the C^α^-H, CH_2_, and CH_3_ vibrational modes of the protein (Figure 1a). The most intense bands, the CH_3_ symmetric and asymmetric stretching modes, appeared at 2883 and 2958 cm^−1^, respectively, together with the Fermi resonance of the methyl group at 2945 cm^−1^, i.e., a resonance between the symmetric methyl stretching and bending overtone. The high obtained intensity of the vibrational modes of the methyl group correlated not only with their large number in the side chains of the protein but also with their highly ordered arrangement at the air–protein interface. The high conformational order of the methyl groups was due to their hydrophobic nature leading them to point more upwards in the direction of the vapor. The contribution of the symmetric (2850 cm^−1^) and asymmetric (2930 cm^−1^) stretching mode, and the Fermi resonance (2910 cm^−1^) of the methylene group, was relatively small. These observations are in good agreement with previous studies at hydrophilic silica surfaces [56] and at the air–water interface [33,57] for globular proteins. The C^α^-H stretching band appeared at 2984 cm^−1^ and showed a large vibrational amplitude in PPP as well as in all chiral polarizations, while it was almost undetectable in the SSP polarization combination. Between 3000 and 3100 cm^−1^, three C–H stretching modes of aromatics, at 3030, 3050, and 3067 cm^−1^, and the overtone of the symmetric bending mode of the NH_3_^+^ groups from the side chains were present. We observed interference effects between the aromatic C–H stretching and the overtones of NH_3_^+^ modes of the side chains (Figure 1a). 

The water molecules contributing to the spectrum in the O–H region were organized in two extreme arrangements: the tetrahedrally coordinated (“ice-like”, ~3200 cm^−1^) and asymmetrically bonded (“liquid-like”, ~3450 cm^−1^) state. At ~3600 cm^−1^, the band belongs to a weakly oriented O–H group, which originates from the interaction with the ester groups of the sidechains of PYP. The narrow bands found at ~3660 and 3700 cm^−1^ can be assigned to the non-hydrogen-bonded, “free” O–H stretching mode of water molecules. The “free” O–H stretching band is usually a narrow, single vibrational mode. Nevertheless, at the air–protein interface in our study, two distinct groups of interfacial water molecules with H atoms protruding into the vapor phase may be found exhibiting different average dipole orientations and O–H bond lengths at the interface.

At chiral polarization combinations (SPP, PSP, and PPS), four C–H stretching modes at 2945, 2970, 2984, and 2998 cm^−1^ appear (Figure 1b). The methylene stretching band was also visible at 2910 cm^−1^ in SPP polarization, which may be a contribution from the C^β^H_2_ groups of sidechains. At 3300 cm^−1^, we observed bands associated with N–H stretches, which were virtually nonexistent at achiral polarizations. This observation of chiral N–H stretching features is in accordance with previously assigned bands with respect to the secondary structure of antiparallel β-sheet or α-helix structures [35]. Around 3200 and 3400 cm^−1^, chiral O–H stretching vibrations were clearly visible as shoulders on the N–H stretching band, which can be attributed to water. This shows that the orientation of the interfacial water molecules was strongly influenced by the polar sidechains of the protein. Previous studies concluded that this water reorientation occurs within an interaction distance of about <10 Å at the air-water interface [58]. This chiral signature of interfacial water is coupled to the chiral N–H stretching modes of the backbone, indicating that the secondary structure of PYP at the air–water interface is well-ordered and the interfacial water molecules that are H-bonded to the backbone or interacting with the polar sidechains are following the ordering of the N–H dipoles. Since the chirality of the C^α^-H group governs the symmetry of the N–H moiety [32], we likely see here the well-ordered β-sheet part of PYP. This β-scaffold motif stands at the end of the protein from the 88th to the 125th amino acid, where most of the side chains (22 out of 38) are polar or charged. The same chiral feature we observed here was previously assigned in the literature to the C–H and N–H groups of the backbone of an antiparallel β-sheet (model peptide of LK_7_), also influencing the orientation of nearby water molecules [58]. From an analysis based on the maximum entropy method (MEM) [59,60] performed on our measured homodyne VSFG spectra (Figure S1), we can assume that the anti-parallel β-sheet part of PYP, i.e., β-scaffold, was more likely oriented parallel to the surface.

### 2.2. VSFG Spectra of Hydrated PEI and PEI+PGA at the Air-CaF_2_ Interface

**Figure 2:**
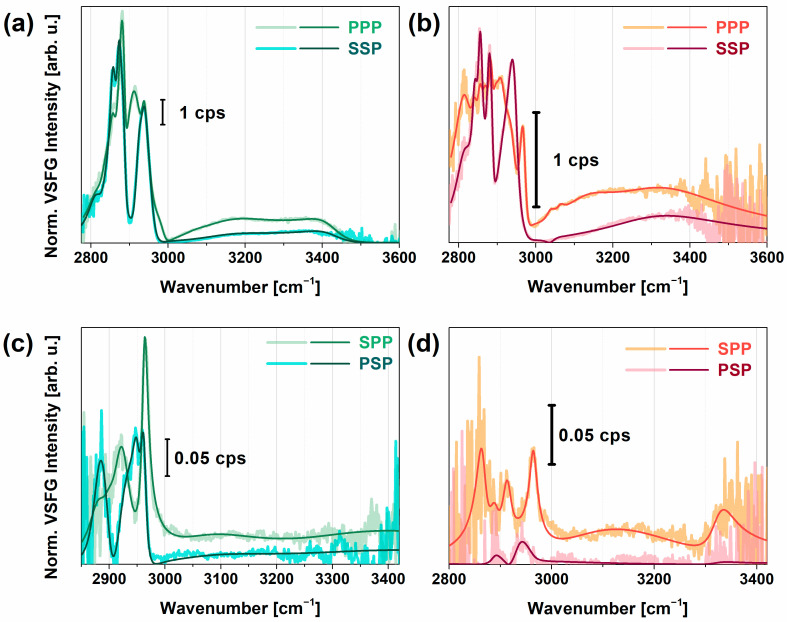
Measured and fitted VSFG spectra of PEI on CaF_2_ (**a**,**c**), and PGA on PEI-CaF_2_ (**b**,**d**). Figures on the top represent the achiral VSFG spectra in PPP and SSP polarizations, figures on the bottom correspond to the chiral SPP and PSP polarization combinations. The fitted curves are based on Equation (1).

Figure 2a–d show achiral (PPP, SSP) and chiral (SPP, PSP) spectra of PEI and PEI+PGA polyelectrolytes at the air–water interface in the spectral range from 2800 to 3600 cm^−1^. While the achiral spectra were dominated by C–H and O–H stretching modes, the chiral features corresponded mostly to C–H vibrational modes. In order to collect more comprehensive information, first, the imaginary part of χ^(2)^ was calculated based on MEM from which the number of vibrational modes and the sign of their amplitudes can be fixed for further analysis. In the next step, each of the normalized VSFG spectra were fitted by a sum of Lorentzian functions based on Equation (1), where the number and sign of the amplitudes of the resonances were taken from the previous MEM analysis. We employed a global fitting procedure (based on Equation (1)) on the VSFG spectra in PPP and SSP, as well as SPP and PSP polarizations. The resulting curves are also shown in Figure 2a–d.

In the achiral spectra obtained for PEI (Figure 2a), characteristic CH_2_ vibrational modes were visible. The bands at 2840, 2856, and 2873 cm^−1^ corresponded to methylene symmetric stretching modes for variously hydrated forms of PEI (i.e., anhydrate, hemihydrate, and dihydrate) for which the asymmetric stretching modes appeared at 2890–2900, 2914, 2925 cm^−1^. Between 2940 and 2990 cm^−1^, two broad features can be extracted with a resonance wavenumber of 2950 and 2980 cm^−1^. Since the bandwidth of both resonances was ~25–40 cm^−1^, we assigned them to the Fermi resonances of the methylene modes. In the O–H stretching range, two broad vibrational bands at 3120 and 3450 cm^−1^ were observed with damping factors of 150 and 100 cm^−1^, respectively. The red-shifted mode at 3120 cm^−1^ showed strong, tetrahedral H-bonding interactions with PEI which can originate from the interaction of interfacial water with the tertiary or secondary amine groups via Coulombic forces. Similar results were observed earlier in the VSFG spectra of various cationic polymers at interfaces [61,62]. The blue-shifted O–H stretching mode at 3450 cm^−1^ corresponded to the asymmetrically bonded, i.e., less than tetrahedral, group of water molecules, most probably near the primary amine groups, since this group has more flexibility in space and this group induces an H-up ordering of water molecules associated with a positive amplitude in the imaginary part of χ² (Figure S2a). In the chiral VSFG spectra of PEI (Figure 2c), C–H stretching bands appeared at 2890, 2900, 2925, and 2950 cm^−1^ suggesting that the hydrated layer contains secondary structural elements in the chain, i.e., double helix conformational parts [63]. Around 3070, 3270, and 3320 cm^−1^, N–H and O–H stretching modes with small amplitudes were observable, which can be linked to the chiral C–H groups. The lower N–H stretching frequency indicates a strong N–H···N hydrogen bond. 

In the achiral VSFG spectra of PGA (Figure 2b) between 2800 and 2950 cm^−1^, several symmetric, asymmetric CH_2_ stretching, and Fermi resonance modes appeared, indicating that the methylene groups were in both trans and cis form. We can observe a very strong band at 2933 cm^−1^ and a doublet at 2952 and 2962 cm^−1^ which can be associated with the C–H stretching modes of the methylene groups. These observed CH_2_ vibrational modes are in good agreement with previous FT-Raman studies [64]. A valley detected at 2991 cm^−1^ can be assigned to the antisymmetric stretch of CH_2_. A weak vibrational mode appeared at 3056 cm^−1^, corresponding to the symmetric bending overtone of the NH_3_^+^ group which was also obtained earlier by using polarized Raman and FTIR spectroscopy on L-glutamine [20]. The appearance of this vibrational mode was more likely due to the charged primary amine groups of branched PEI which interacted with the side chains of PGA. For interfacial water, the lower-frequency vibrational band around 3150 cm^−1^ (FWHM ~ 110 cm^−1^) corresponded to strongly H-bonded interfacial water; namely, it revealed the Coulombic–ionic interaction between the COO^─^ group and H···O–H. In Figure S2, this band showed a negative amplitude in the imaginary part, suggesting an H-down orientation on the top of PGA. The broad vibrational band at ca. 3350 cm^−1^ (FWHM ~200 cm^−1^) was the overtone of the OH bending mode. Such a broad feature comes from a broad orientational distribution of interfacial water [65], which is most probably embedded in and between the backbone of PGA. Near 3500 cm^−1^, a Fano-shape resonance was shown, which can be linked to the stretching overtone of the C=O group. Since the stretching frequency of this group is very sensitive to the intramolecular H-bonding, we may also see here the interaction of some C=O groups with water molecules.

At chiral polarizations (Figure 2d), C–H vibrational bands of PGA were identified at 2890, 2934, and 2970 cm^−1^. We attributed the first and third vibrational bands to the out-of-plane and in-plane C^α^-H stretching modes, respectively, governing chirality in amino acids. This doublet was also obtained in the Raman spectra of alcohols [21]. Above 3000 cm^−1^, a very weak signal with broad bandwidth was detected in both SPP and PSP polarizations, suggesting that PGA does not form well-ordered β-sheet or α-helical arrangements since the characteristic chiral N–H stretch at ~3300 cm^−1^ was missing. Instead, PGA most probably lies parallel to the surface of PEI in a random coil structure, given that the chiral N–H stretching signal is forbidden in random coil and disordered structures [35]. We found that the overall VSFG signal decreased when PGA was adsorbed on PEI, which was most likely due to destructive interference between the methylene modes of PEI and PGA.

Since the homogeneity of the PEI and PEI+PGA layers can be improved by creating several pairs of oppositely charged layers [66,67,68], we studied how the VSFG spectra of the topmost layer of PGA changed when 0.5 versus 6.5 pairs of layers were built up. The results are summarized in Figure S3. In each of the applied polarization combinations (PPP, SSP, SPP), we observed a much (factor of two) higher signal for 6.5-pair layers due to the higher surface homogeneity. Importantly, the red-shifted O–H stretching mode in each polarization showed a higher signal which can be attributed to a well-ordered interfacial water structure near the charged side chains of amino acids. 

### 2.3. PYP Adsorption at Air-Polyelectrolyte Interfaces

**Figure 3:**
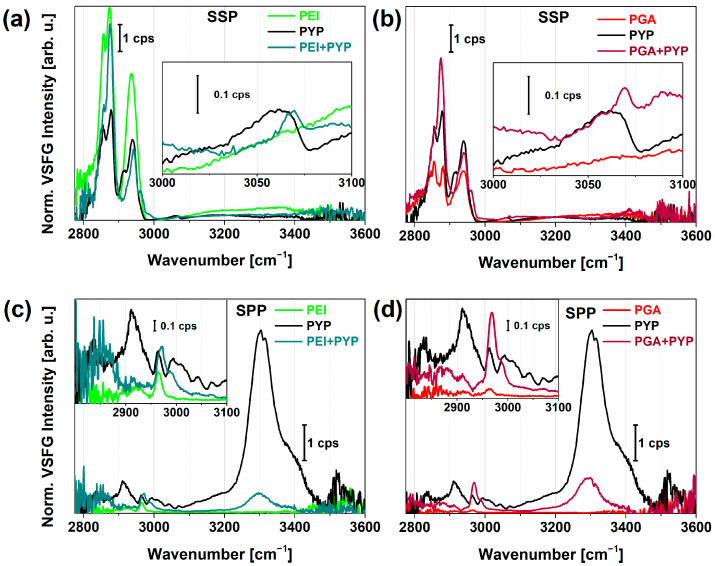
Normalized VSFG spectra of photoactive yellow protein (PYP) adsorbed on PEI (**a**,**c**), PGA (**b**,**d**) polyelectrolyte layers prepared by the LbL method. For comparison, the corresponding spectrum of PYP film (black) is also shown. Panel (**a**,**b**) and (**c**,**d**) show achiral and chiral VSFG spectra, respectively.

Figure 3a–d show the VSFG spectra of adsorbed PYP on PEI and PEI+PGA layers in the range of 2800 and 3600 cm^−1^ obtained in SSP and SPP polarization combinations. For comparison, the spectra acquired for the polyelectrolyte layers and the hydrated PYP film separately are also plotted. As shown in Figure 3, the adsorption of PYP on the polyelectrolyte layers of PEI and PEI+PGA led to significant spectral changes. The C^α^-H stretching feature showed up as a shoulder at 2980 cm^−1^ in both achiral and chiral polarizations. This was observed previously by other groups on various types of proteins (e.g., LK_7_β, pepsin). While strong stretching modes of methylene groups were observed from the polyelectrolytes, almost every C–H stretching mode was shifted when PYP was adsorbed on the polyelectrolyte surfaces. These shifts were due to the vibrational modes of methyl groups from the nonpolar side chains of PYP, similar to what was shown in the case of hydrated PYP on CaF_2_. The vibrational modes centered at 2860, 2885, 2915, and 2945 cm^−1^ were previously assigned (in the case of hydrated PYP) to the CH_2_ and CH_3_ symmetric stretch, CH_2_ asymmetric stretch, and the Fermi resonance of the CH_3_ group, respectively. Between 3000 and 3100 cm^−1^, characteristic aromatic C–H stretching modes interfered with the symmetric bending overtone of the NH_3_^+^ group derived from PYP side chains. The VSFG spectra obtained for PYP and adsorbed PYP on both surfaces showed a marked difference. The bands at 3030, 3050, and 3070 cm^−1^ appeared in chiral polarization clearly without interference for each PYP sample, while in achiral polarizations, the adsorbed PYP showed a valley at 3030 cm^−1^ with narrow bands at 3070 and 3085 cm^−1^ due to the interference between the aromatics C–H stretching with the amide B mode. 

**Figure 4:**
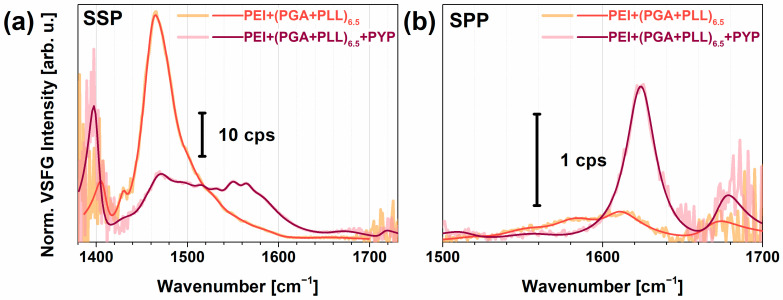
Achiral (**a**) and chiral (**b**) spectra of (PGA-PLL)_6.5_ (light red) and (PGA-PLL)_6.5+_PYP (dark red) in the amide I region. The relative humidity of the films was ~80%.

In the chiral spectra in Figure 3c,d, the adsorption of PYP on both PEI and PGA lead to the emergence of characteristic bands at 2943, 2958, 2976, and 2990 cm^−1^, in accordance with the presence of C^α^-H and CH_3_ groups of various amino acids in PYP. The valley at 2958 cm^−1^ and the local peak at 2976 cm^−1^ can be attributed to C^α^-H stretching. These bands showed opposite signs in the imaginary χ² spectra (Figure S2), suggesting that the C^α^-H stretch exhibited the same chirality and orientation as the N–H stretch at ~3300 cm^−1^. From this information, we can conclude that these chiral vibrational modes can be connected to the antiparallel β-sheets with hydrogen bonds between C=O and H–N, which were lying on the plane of the surface. The appearance of the spectral shoulder at 2990 cm^−1^ is an indication of a C^α^-H bond from other amino acids, most often assigned to lysine [32,57]. In the structure of the β-scaffold motif of PYP, lysine can be found in the highest number. The β-sheet structure was also supported by the well-ordered N–H stretching and the presence of a strong chiral amide I vibrational mode (Figure 4b). We observed only small differences in the VSFG spectra of PYP adsorbed on PEI versus PGA. However, the higher overall VSFG intensity obtained for PYP on PGA than on PEI suggests that a larger amount of PYP was adsorbed on the PGA surface, possibly due to the random coil structure of PGA facilitating better PYP adhesion. The vibrational bands corresponding to C^α^-H and N–H stretching modes were red-shifted for PGA+PYP, indicating stronger interaction, i.e., shorter bond lengths. At 2990 cm^−1^, the sign of the vibrational mode was opposite, positive for PEI+PYP and negative for PGA+PYP.

Next, the effect of the improved structural homogeneity of the topmost PGA layer in multilayer stacks on the adsorption of PYP was studied. Investigations of the multilayer structures using atomic force microscopy (AFM) revealed a very homogeneous surface for PEI+(PGA+PLL)_6.5_ and an increase in surface roughness upon PYP adsorption (Figure S4). A densely packed surface was clearly discernible when PYP was adsorbed onto the PGA layer with a concomitant decrease in interfacial stiffness. This decrease is consistent with the formation of a relatively soft protein layer, which was found to be very homogeneous over several microns. In a separate set of experiments, we also cross-checked the stability of the rehydrated PEI+(PGA+PLL)_6.5_+PYP film, and only minor structural changes were obtained in the C–H stretching region (Figure S6).

In Figure 4, the acquired VSFG spectra of the polyelectrolyte multilayer, PEI+(PGA+PLL)_6.5_, with and without PYP adsorption is shown in the vibrational region between 1380 and 1720 cm^−1^, and between 1500 and 1700 cm^−1^ for achiral and chiral polarization combinations, respectively. This region is suitable to study not only the amide I and II modes, but also the side-chain vibrations. The tables in the supplementary material summarize the assignments of the achiral (Tables S1 and S3) and chiral (Tables S2 and S4) vibrational modes of the multilayer structures without and with PYP, respectively, obtained from fitting Lorentzian line profiles based on Equation (1) onto the spectra. 

At 1402 cm^−1^, the symmetric COO^─^ stretched from aspartate and glutamate units can be seen in the PYP spectrum, while it shifted to 1408 cm^−1^ on the polymer interface (Figure 4a). Without PYP, huge CH_2_ deformation bands of polymers appeared at 1425–1475 cm^−1^, which were more intense and can be seen at lower frequencies (i.e., 1425 cm^−1^) when next to a C=O moiety. Upon PYP adsorption, these deformation modes decreased and broadened, suggesting that the methylene groups of PYP have random orientational distribution. The asymmetric deformation mode of CH_3_ was around 1445–1480 cm^−1^, while the symmetric and asymmetric bending modes of the NH_3_^+^ group lay at 1527 and 1625 cm^−1^, respectively, and each one was observed for both with and without PYP. When PGA was the topmost layer, the NH_3_^+^ bending modes more likely appeared from the Lys layer under the PGA. For PYP, the ring mode near 1517 cm^−1^ was detected, which is usually very characteristic in protein absorption spectra from the Tyr side chain. At 1583 cm^−1^, a band appeared in chiral polarization when the topmost layer was PGA, which can be assigned to asymmetric COO^─^ stretching. For PYP, bands at 1590 and 1610 cm^−1^ were detected. The lower frequency band belonged to the COO^─^ stretching mode of Glu/Asp side chains that were red-shifted during the interaction of PYP with the PGA surface. Since the stretching frequency of COO^─^ moiety is very sensitive to the local environment, it may shift ±40–60 cm^−1^ [69]. A chiral band at 1610 cm^−1^ was found both with and without PYP due to a bifurcated H-bonding to −COOH groups on the protonated Glu side chain, which reflects very strong H-bonding [70,71]. Interestingly, ROA studies of polylysine, polyglutamic acid, and some proteins also showed a negative/positive signal at 1610/1626 cm^−1^ for β-sheet structures [72], which were later linked to the formation of β_2_-fibrils [73].

In the achiral spectra, the weak vibrational bands at 1640 cm^−1^ and 1665 cm^−1^ can be assigned to the amide I B_2_ and B_1_ mode of the antiparallel β-sheets, respectively. In the chiral spectrum of PYP, these amide I modes were enhanced and red-shifted, while the amide II band near 1560 cm^−1^ is also characteristic. Our observations on the chiral N–H stretching together with the chiral amide I and II modes confirmed that chiral VSFG spectroscopy is capable of determining the type of secondary structure with the highest abundance of PYP, i.e., β-scaffold in this case. 

**Figure 5:**
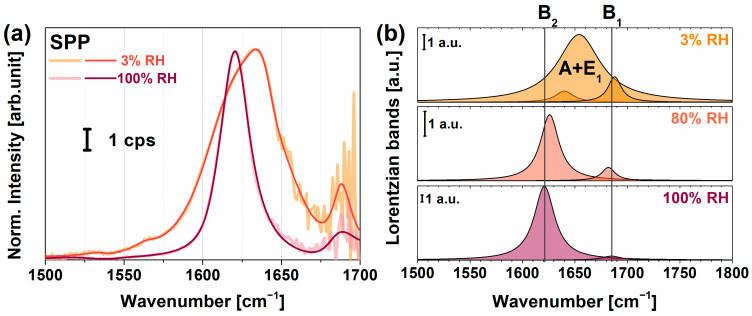
(**a**) Chiral VSFG spectra of PEI+(PGA+PLL)_6.5_+PYP multilayer in different relative humidity environments, orange corresponding to low (3%) and dark red corresponding to high (100%) relative humidity. (**b**) Lorentzian components of the amide I modes of PYP at 3%, 80%, and 100% relative humidity (RH) based on the fitting parameters listed in Tables S4–S6. The B_1_ and B_2_ modes correspond to β-sheet structures, and the A and E_1_ mode belong to α-helices.

Figure 5a shows the chiral spectra of PYP adsorbed on the PEI+(PGA+PLL)_6.5_ multilayer structure in the spectral range from 1400 to 1700 cm^−1^ at a relative humidity of 3% and 100%. Tables S5 and S6 contain the assignments of the corresponding vibrational modes of the multilayer structures without and with PYP, respectively, obtained from fitting based on Equation (1) onto the spectra. The characteristic amide I B_1_ and B_2_ modes at high humidity were very narrow with damping factors lying between 8 and 11 cm^−1^, respectively, indicating a narrow orientational distribution of the peptide bonds in the β-scaffold part of PYP. The B_2_ mode of the β-sheet structure lay at 1621 cm^−1^, where the low frequency value can be explained by very strong H-bonds. When the humidity decreased, no broadening of the chiral B_1_ and B_2_ vibrational modes was observed. However, the B_2_ mode blue-shifted to 1640 cm^−1^ (almost the highest frequency attainable to a B_2_ band) and a new peak arose at 1654 cm^−1^, which we assigned to the amide I mode, i.e., A and E_1_, for an α-helix secondary structure. Figure 5b visualizes the corresponding changes of the amide I modes at various humidity conditions based on the fitted parameters in Tables S4–S6. Yan and co-workers obtained no chiral amide I signal for rhodopsin, pHLIP, and LK_α_14 model systems [35], suggesting that the chiral amide I mode was silent for helical structures. In contrast, Ishibashi’s group found a small but significant chiral amide I band for BSA with a secondary structural content of 67% α-helix and 10% β-turn [33]. The fact that we could resolve the shoulder at 1654 cm^−1^ was made possible by the high spectral resolution (~3 cm^−1^) of our home-built VSFG spectrometer and its high sensitivity thanks to the employed 100-kHz laser system. Since globular proteins easily undergo denaturation, we expect that PYP loses the hydrogen shell at low humidity. Therefore, a partial loss of its tertiary structure was likely detected, which changed the orientation of α-helixes and β-sheets and also weakened their H-bond structure. Detailed calculations of molecular orientation for polyelectrolyte-PYP interfaces are in progress.

## 3. Materials and Methods

### 3.1. Sample Preparation

**Figure 6:**
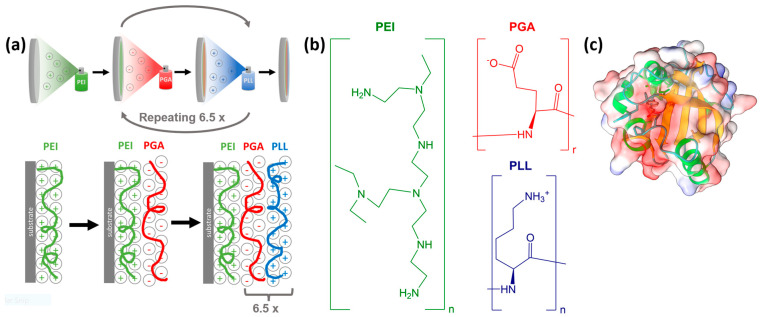
(**a**) Schematic illustration of the spray-assisted LbL assembly on CaF_2_ substrate using oppositely charged polyelectrolytes. (**b**) The chemical structures of polyethyleneimine (PEI), poly-l-glutamic acid (PGA), and poly-L-lysine (PLL). (**c**) Secondary structural elements and the electrostatic potential distribution of PYP. The protein structure was generated with ChimeraX [74]. For the VSFG measurements, PYP was adsorbed on the topmost layer of PEI, PEI-PGA, and PEI-(PGA-PLL)_6.5_.

To prepare the polyelectrolyte layers, we used branched polyethyleneimine, poly-l-glutamic acid, and poly-L-lysine by utilizing the layer-by-layer method. All three polyelectrolytes were purchased from Sigma-Aldrich and had a molecular weight of 600,000–1,000,000 (branched PEI solution, concentration of ~50% in H_2_O), 50,000–100,000 (PGA sodium salt), and >30,000 (PLL hydrochloride). The applied PEI stock solution had a concentration of 5 mg/mL, while the concentration of PGA and PLL was 1 mg/mL. First, PEI was sprayed onto an oxygen plasma-cleaned CaF_2_ window to fully coat the substrate and provide a base for subsequent layers. The deposited PEI layer was left for a minute, the residue was then thoroughly washed off with distilled water. In the following step, a layer of PGA was added and left to adsorb for 20 s with the residue before being washed off. The PGA layer was followed by adding and similarly washing a layer of PLL. We continued to add PGA and PLL layers up to a total of six-and-a-half pairs, as this resulted in a very homogeneous surface [66,67,68]. A layer of PYP was added to the topmost polyelectrolyte layer by pipetting the PYP stock solution of 0.28 mM on top and letting it set for 5 min, then washing it off similarly to the case of the previous layers. After preparation, the samples were left to equilibrate for at least 10 min before collecting VSFG spectra. The measurements were carried out on PEI, PEI+PGA, and six-and-a-half pairs of PGA+PLL layers on PEI (denoted as PEI+(PGA+PLL)_6.5_). Additionally, PYP was adsorbed on top of PEI, PEI+PGA, and PEI+(PGA+PLL)_6.5_ samples. The LbL assembly and the chemical structures of the applied polyelectrolytes are summarized in Figure 6. While repeating the measurements, the samples were held in a hydrated state by keeping them in a >80% relative humidity environment.

### 3.2. Vibrational Sum-Frequency Generation (VSFG)

The VSFG setup was described in detail elsewhere [53,54]. Here, only a brief account is given. The pump laser employed in the VSFG spectrometer was a Yb:KGd(WO_4_)_2_ laser oscillator-amplifier system operating at a center wavelength of 1028 nm with a repetition rate of 100 kHz. The pump pulses were split into two parts. One part was forwarded into a home-built spectral compressor which generated narrowband visible pulses at 514 nm, while the other part of the beam was used to generate tunable MIR laser pulses in the spectral ranges of 2800–3800 cm^−1^ and 1400–1700 cm^−1^ via optical parametric amplification. On the path of the infrared pulses, a home-made purging-enclosure system was used to minimize absorption by atmospheric water vapor and CO_2_. The energy of the visible pulses was kept at 4 µJ per pulse, while the pulse energies of the mid-infrared pulses centered at wavenumbers of 1267, 2980, and 3455 cm^−1^ were 0.2, 0.7, and 0.7 µJ, respectively. All listed pulse energies refer to the incident values on target. The visible and infrared pulses were focused onto the sample and overlapped temporally and spatially. The angles of incidence for the pulses were 68° and 57°, respectively. The VSFG signal was collected by a spectrometer equipped with a Peltier-cooled, deep-depletion charge-coupled device. The spectral resolution of the VSFG spectrometer was ~3 cm^−1^. 

The polarization for the input beams was controlled by using zero-order half-waveplates, while an additional polarizer with a half-waveplate was employed at the entrance of the spectrometer for the SFG beam. Spectra in the C–H, N–H, and O–H stretching regions were collected in PPP, SSP, SPP, PSP, and PPS polarization combinations (the order of polarizations corresponds to SFG, visible and infrared beams, respectively), while spectra in the amide I region were collected in SSP and SPP polarization combinations. The acquisition times ranged from 10 s to 120 s—shorter times were applied in the C–H, N–H, and O–H stretch regions, while longer times were used in the amide I region. All measurements were repeated at least 3 times and at different sample positions to minimize and account for the effect of the environment on the acquired spectra. The measurements were carried out at room temperature (23 °C) and controlled relative humidity. 

The VSFG spectra were frequency calibrated using a 50-µm-thick polystyrene film, which was inserted into the MIR beam. Difference spectra were calculated by subtracting the background spectrum from each measurement (the spectrum without infrared excitation). To convert spectral intensity into count-per second, the difference spectra were divided by the acquisition time. The non-resonant spectrum measured at a silver surface was normalized to one and was then corrected by multiplying it by the measured infrared intensity at the sample’s surface for the absolute comparison of the different spectral regions. The VSFG difference spectra were then normalized by this corrected non-resonant spectrum. Finally, the normalized spectra were fitted by the sum of Lorentzian functions that describe the resonant part of the obtained signal and a non-resonant additional part using the following equation:

(1) $$I_{VSFG}\;\left(\omega \right)\propto {\left|P_{NR}e^{i\Phi }+\sum_{i=1}^{\nu }\frac{Q_{\upsilon }}{\omega -\omega_{\nu }-i\Gamma_{\nu }}\right|}^{2},$$

where *Q_ν_*, *ω_ν_*, and *Γ_ν_* are the strength, frequency, and damping factor of the *v*th Lorentzian peak. The first element of the sum accounts for the non-resonant contribution with amplitude *P_NR_* and phase *Φ*, making it possible to describe both constructive and destructive interference.

### 3.3. Atomic Force Microscopy (AFM)

AFM images were obtained on a Bruker/JPK NanoWizard 4 operating in Quantitative Imaging (QI) mode. In this mode, force spectra were collected at every point in a given image area, from which several nanomechanical parameters such as height, stiffness, and adhesion were extracted. The height was estimated from a setpoint force chosen during the experiment, while the stiffness was estimated as the slope of the approach curve. The images were obtained using Nanosensors PPP-NCH probes.

## 4. Conclusions

Chiral and achiral VSFG spectroscopy was performed to study the adsorption properties of photoactive yellow protein on positively and negatively charged, self-assembled polyelectrolyte surfaces and layer stacks. We demonstrated that homodyne VSFG spectroscopy is a viable technique for the structural study of nm-scale multilayers, where the orientational information is extracted by employing the maximum entropy method (MEM) and the standard global fitting procedure on the VSFG spectrum in conjunction. We found that the multilayer stack leads to a much more homogenous top layer when 6.5 layers are employed than in the 0.5-layer case. Structural homogeneity was revealed by increasing vibrational band amplitudes and decreasing bandwidths which were further confirmed by AFM studies. Our data suggest that while PEI shows helical structural elements, PGA forms mostly random coil arrangement with a small amount of β_2_-fibril structure at the interface at physiological pH, and these structures do not change when more pairs of layers are applied. 

Due to our high spectral resolution, the C^α^, C^β^, and C^γ^ signals can also be spectrally resolved during the buildup of polymer layer structures, which can be used to follow the cis–trans changes of methylene groups. If the protein is adsorbed, the methylene modes become less informative as a result of interference effects. Nevertheless, the methyl groups become well-ordered in this case due to the air-layer interface giving a strong CH_3_ stretch signal. Acquiring chiral spectra revealed details about the β-scaffold portion of PYP, while the achiral signal of the hydrated PYP layer was not specific to the protein. However, upon PYP adsorption on charged surfaces, the C^α^-H, aromatics C–H, and side-chain N–H vibrational bands, characteristic of proteins, also appeared in achiral polarization combinations, making achiral signals also surface-specific. 

Chiral VSFG spectra of adsorbed PYP contain a wealth of information: the homodyne N–H stretch signal showed a redshift for negatively charged PGA+PYP compared to PEI+PYP, which was also confirmed by the spectra extracted using the MEM procedure. The relative redshift in the PGA+PYP case suggests that PYP keeps its tertiary structure to a higher degree when interacting with PGA than with PEI. The AFM characterization showed that an intact PYP layer was formed on the PGA-terminated surface, as evidenced by an increase in surface roughness and a homogenous reduction in interfacial stiffness. We also found that both the chiral and achiral VSFG spectra obtained in the spectral region of 1400–1700 cm^−1^ exhibited significant differences when PYP adsorbed at the interface which can be linked to the amide I and II modes and side chain vibrations. The changes in the amide I and II bands suggest that PYP loses its external hydrogen shell at low humidity in spite of a stable beta-sheet secondary structural motif. At the same time, some minor denaturation, i.e., partial loss of tertiary structure, was also detectable. Our results corroborate that chiral VSFG spectroscopy can determine the secondary structure of proteins which has the highest abundance and is additionally very sensitive to the tertiary structure of proteins.

## Data Availability

Data are available upon request.

## Supplementary Materials

The following supporting information can be downloaded at: https://www.mdpi.com/article/10.3390/molecules28104077/s1, Figure S1: Imaginary part of the chiral VSFG spectra of PYP in the C–H, N–H, and O–H stretch region calculated from the observed SPP spectrum via the MEM algorithm.; Figure S2: Calculated imaginary part of χ^(2)^ of PEI and PEI+PYP (a,c), and PGA and PGA+PYP (b,d) in SSP and SPP polarizations, respectively.; Figure S3: Achiral (a,b) and chiral (c) VSFG spectra of one pair of PEI+PGA (light red) and PEI+(PGA+PLL)_6.5_ (dark burgundy) multilayers in the C–H, N–H, and O–H stretch region. The topmost layer contains PGA in each case. Table S1: Vibrational mode assignments and corresponding VSFG wavenumbers, spectral widths, and amplitudes of PEI+(PGA+PLL)_6.5_, i.e., topmost layer is PGA, in the spectral region between 1400 and 1700 cm^−1^ at a relative humidity of 80%. Table S2: Vibrational mode assignments and corresponding VSFG wavenumbers, spectral widths, and amplitudes of PEI+(PGA+PLL)_6.5_+PYP, i.e., topmost layer is PYP, in the spectral region between 1400 and 1700 cm^−1^ at a relative humidity of 80%. Table S3: Chiral vibrational mode assignments and corresponding VSFG wavenumbers, spectral widths, and amplitudes of PEI+(PGA+PLL)_6.5_, i.e., topmost layer is PGA, in the spectral region between 1500 and 1700 cm^−1^ at a relative humidity of 80%. Table S4: Chiral vibrational mode assignments and corresponding VSFG wavenumbers, spectral widths, and amplitudes of PEI+(PGA+PLL)_6.5_+PYP in a relative humidity of 80% in the spectral region between 1500 and 1700 cm^−1^. Table S5: Vibrational mode assignments and corresponding VSFG wavenumbers, spectral widths, and amplitudes of PEI+(PGA+PLL)_6.5_+PYP in a relative humidity (RH) of 3% in the spectral region between 1500 and 1700 cm^−1^. Table S6: Vibrational mode assignments and corresponding VSFG wavenumbers, spectral widths, and amplitudes of PEI+(PGA+PLL)_6.5_+PYP in a relative humidity (RH) of 100% in the spectral region between 1500 and 1700 cm^−1^. Figure S4: AFM images of PEI+(PGA+PLL)_6.5_ multilayers without and with PYP. Figure S5: Histogram of stiffness values from AFM images without and with PYP. Figure S6: Structural stability of a PEI+(PGA+PLL)_6.5_+PYP film over time, i.e., 1 day and 50 days after film assembly.

## Sample Availability

Samples from the authors are available upon request.
