# Supplementary material for: Photoactive Yellow Protein Adsorption at Hydrated Polyethyleneimine and Poly-l-Glutamic Acid Interfaces
Source: Molecules. 2023 May 13;28(10):4077. doi: 10.3390/molecules28104077 (PMC10223613; doi:10.3390/molecules28104077)
Supplement: Supplementary file 1 [file molecules-28-04077-s001.zip › molecules-2362230-supplementary.pdf]

Supplementary Materials

Photoactive yellow protein adsorption at hydrated polyethyleneimine and poly-L-glutamic acid interfaces

Szilvia Krekic[1,2,3], Mark Mero[4], Michel Kuhl[1,5], Kannan Balasubramanian[1,5], András Dér[2], and Zsuzsanna Heiner[1]*

[1] School of Analytical Sciences Adlershof, Humboldt-Universität zu Berlin, Berlin, Germany
[2] Institute of Biophysics, Biological Research Centre, Szeged, Hungary
[3] Doctoral School of Multidisciplinary Medical Sciences, University of Szeged, Szeged, Hungary
[4] Max Born Institute for Nonlinear Optics and Short Pulse Spectroscopy, Berlin, Germany
[5] Department of Chemistry and IRIS Adlershof, Humboldt-Universität zu Berlin, Berlin, Germany

*Correspondence: heinerzs@hu-berlin.de

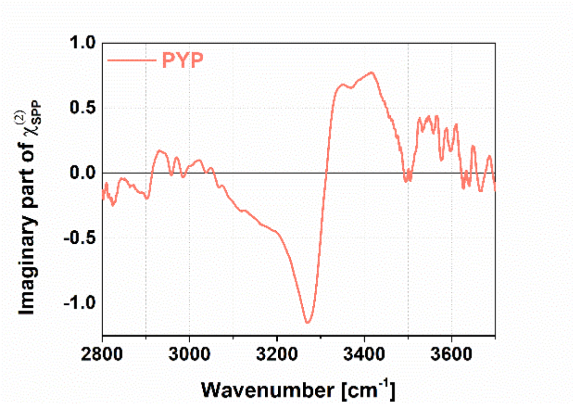

Figure S1. Imaginary part of the chiral VSFG spectra of PYP in the C-H, N-H, and O-H stretch region calculated from the observed SPP spectrum via the MEM algorithm.

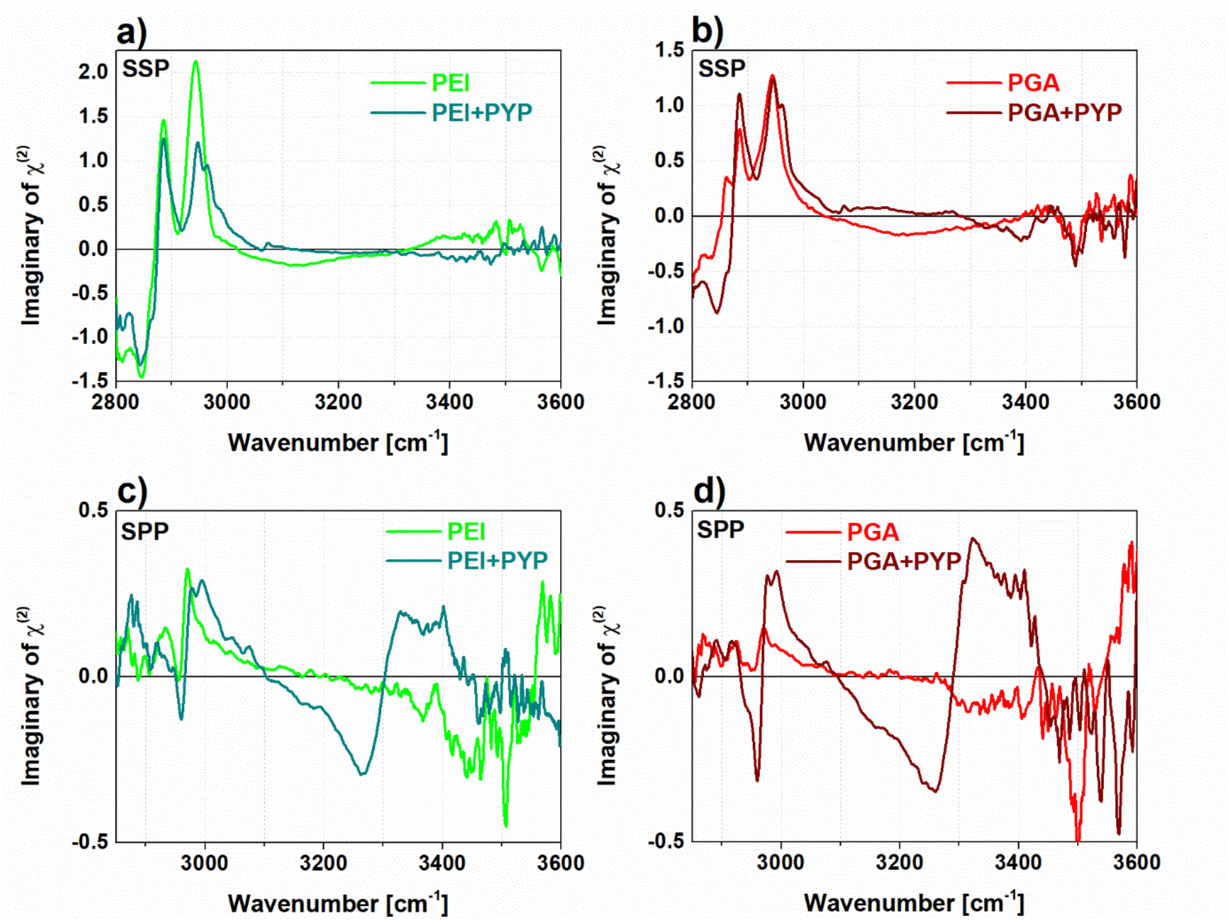

Figure S2. Calculated imaginary part of $\chi^{(2)}$ of PEI and PEI+PYP **(a, c)**, and PGA and PGA+PYP **(b, d)** in SSP and SPP polarizations, respectively.

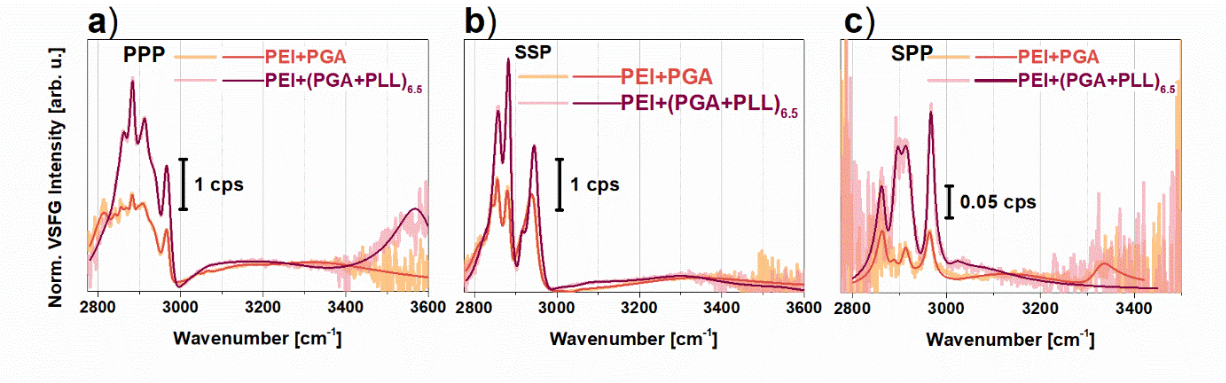

Figure S3. Achiral **(a, b)** and chiral **(c)** VSFG spectra of one pair of PEI+PGA and PEI+$(PGA+PLL)_{6.5}$ multilayers in the C-H, N-H, and O-H stretch region. The topmost layer contains PGA in each case.

Table S1. Vibrational mode assignments and corresponding VSFG wavenumbers, spectral widths, and amplitudes of PEI+(PGA+PLL)$_{6.5}$, i.e., topmost layer is PGA, in the spectral region between 1400 and 1700 cm^{-1} at a relative humidity of 80%.

**Band assignments, achiral (SSP)**	**ω [cm^{-1}]**	**Γ**	**Q**
ν_S COO$^-$	1408	8	16
δ CH$_2$ / ν C-N	1430	5	3.9
δ CH$_2$	1463	13.6	53.2
δ CH$_2$	1475	17.4	65.7
δ CH	1499	19.8	29
δ_S NH$_3^+$ (from Lys layer, or end terminal)	1523	16	11
ν_{AS} COO$^-$	1550	14	2
δ NH$_2$	1597	15	-3.7
ν C=O and contribution from δ_{AS} NH$_3^+$	1646	28	6.9

Table S2. Vibrational mode assignments and corresponding VSFG wavenumbers, spectral widths, and amplitudes of PEI+(PGA+PLL)$_{6.5}$+PYP, i.e., topmost layer is PYP, in the spectral region between 1400 and 1700 cm^{-1} at a relative humidity of 80%.

**Band assignments, achiral (SSP)**	**ω [cm^{-1}]**	**Γ**	**Q**
ν_S COO$^-$	1400	8	33
δ CH$_2$	1430	20	17.8
δ CH, CH$_2$, and CH$_3$	1455	8	1
δ_{AS} CH$_3$	1470	19	40.7
ν C-C / δ CH (Tyr, Trp, Phe)	1497	26	60
ν C-C (Tyr-OH)	1516	13	11.4
δ_S NH$_3^+$ (Lys)	1531	16	11
Amide II / ν_{AS} COO$^-$ (Glu, Asp)	1548	14	26.1
Amide II / ν_{AS} COO$^-$ (Glu, Asp)	1564	8.5	5.8
ν_{AS} COO$^-$ *	1578	21	24.5
Amide I	1640	20	1
Amide I	1665	25	13.1

Table S3. Chiral vibrational mode assignments and corresponding VSFG wavenumbers, spectral widths, and amplitudes of PEI+(PGA+PLL)$_{6.5}$, i.e., topmost layer is PGA, in the spectral region between 1500 and 1700 cm^{-1} at a relative humidity of 80%.

**Band assignments, chiral (SPP)**	**ω [cm^{-1}]**	**Γ**	**Q**
δ_S NH_3^+ (Lys)	1521	14.6	1
ν_{AS} COO^- *	1554	20	4.1
ν_{AS} COO^- *	1583	20	7.1
–COOH / δ NH_2	1609	14.4	4.8
ν C=O	1668	13.9	4.1

* Since the stretching frequency of COO^- group very sensitive to the environment, may shift +/- 40 cm^{-1}

Table S4. Chiral vibrational mode assignments and corresponding VSFG wavenumbers, spectral widths, and amplitudes of PEI+(PGA+PLL)$_{6.5}$+PYP in a relative humidity of 80% in the spectral region between 1500 and 1700 cm^{-1}.

**Band assignments at RH 80%, chiral (SPP)**	**ω [cm^{-1}]**	**Γ**	**Q**
ν C-C (Tyr, Trp, Phe)	1516	14.5	-3.6
Amide II / ν_{AS} COO^- (Glu, Asp)	1554	19.1	-4.2
ν_{AS} COO^- (Glu, Asp)	1588	21.6	-3.3
–COOH / δ NH_2 (Gln, Asn) / ν C-C (Tyr, Trp)	1612	15.8	-9.3
Amide I B2 mode for β-sheet	1626	10.7	17.5
Amide I B1 mode for β-sheet, ν C=O (Glu, Asp)	1682	7.7	5.7

Table S5. Vibrational mode assignments and corresponding VSFG wavenumbers, spectral widths, and amplitudes of PEI+(PGA+PLL)$_{6.5}$+PYP in a relative humidity (RH) of 3% in the spectral region between 1500 and 1700 cm^{-1}.

**Band assignments at RH 3%, chiral (SPP)**	**ω [cm^{-1}]**	**Γ**	**Q**
ν C-C (Tyr, Trp, Phe)	1510	30	-2
δ_S NH_3^+ (Lys)	1533	9.4	-2.2
Amide II / ν_{AS} COO^- (Glu, Asp)	1564	12.7	-3.9
ν_{AS} COO^- (Glu, Asp)	1590	24.5	-9
–COOH / δ NH_2 (Gln, Asn) / ν C-C (Tyr, Trp)	1610	25	-60.1
Amide I B2 mode for β-sheet	1640	10.3	9
Amide I for α-helix	1654	24.7	52.7
Amide I B1 mode for β-sheet, ν C=O (Glu, Asp)	1688	7.6	10

Table S6. Vibrational mode assignments and corresponding VSFG wavenumbers, spectral widths, and amplitudes of PEI+(PGA+PLL)$_{6.5}$+PYP in a relative humidity (RH) of 100% in the spectral region between 1500 and 1700 cm^{-1}.

**Band assignments at RH 100%, chiral (SPP)**	**ω [cm^{-1}]**	**Γ**	**Q**
δ_S NH_3^+ (Lys) / ν C-C (Tyr, Trp, Phe)	1522	16	2.8
Amide II / ν_{AS} COO^- (Glu, Asp)	1545	8	-0.5
ν_{AS} COO^- (Glu, Asp)	1590	13.9	-0.8
–COOH / δ NH_2 (Gln, Asn) / ν C-C (Tyr, Trp)	1605	12.5	-6.6
Amide I B2 mode for β-sheet	1621	11.9	39.4
Amide I B1 mode for β-sheet, ν C=O (Glu, Asp)	1685	11.3	8.9

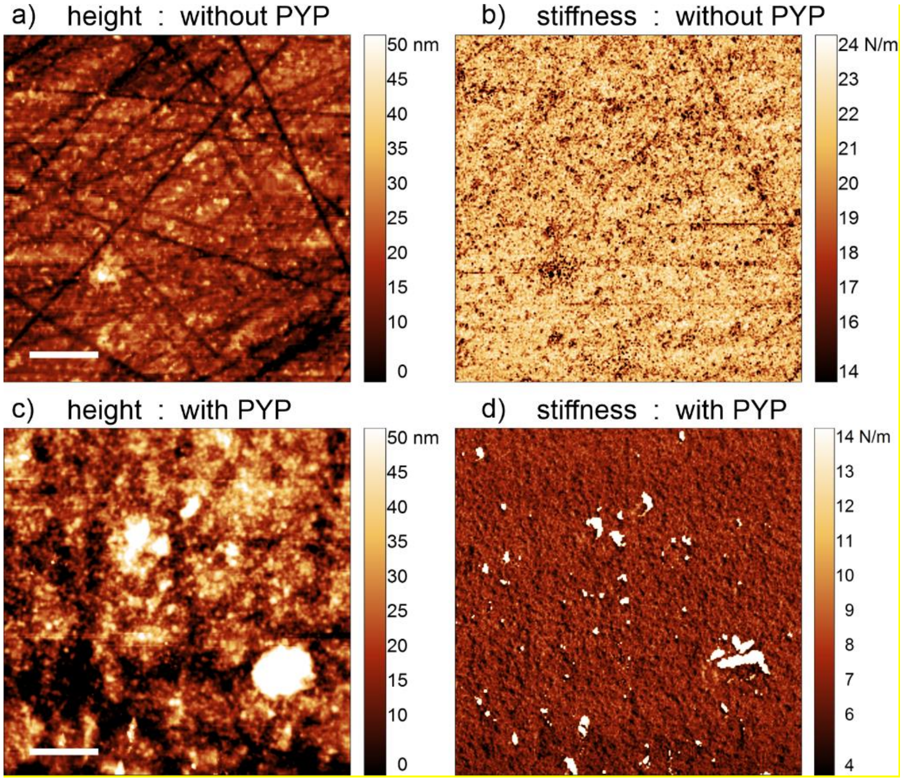

Figure S4. Surface characterization of (a,b) PEI+(PGA+PLL)$_{6.5}$ and (c,d) PEI+(PGA+PLL)$_{6.5}$+PYP multilayers using Quantitative Imaging mode AFM. Before deposition of PYP, we observe a surface topography typical for CaF_2 substrates (a). After coating the surface with the protein, a coarse surface is observable (c). The crack-like lines seen in (a) are not observable anymore, suggesting that the surface is indeed covered by a molecular layer. After coverage the roughness appears to be slightly higher. However, the height range is in the same order as before protein adsorption. This suggests that the protein indeed forms a conformal layer on the PGA-terminated CaF_2 surface. The presence of the protein layer is further attested by the stiffness images (b,d), which plot the slope of the AFM approach curve as a

function of spatial position. The stiffness is higher (range: 14 – 24 N/m) on the PGA-terminated surface (b) in comparison to the surface after protein film formation (d, range: 4 – 14 N/m). A protein layer signifies a soft surface where the stiffness is expected to be lower. Furthermore, the stiffness is quite homogenous over the entire surface (see histograms in fig. S5) confirming the formation of an intact protein layer. Scale bar is 2 μm.

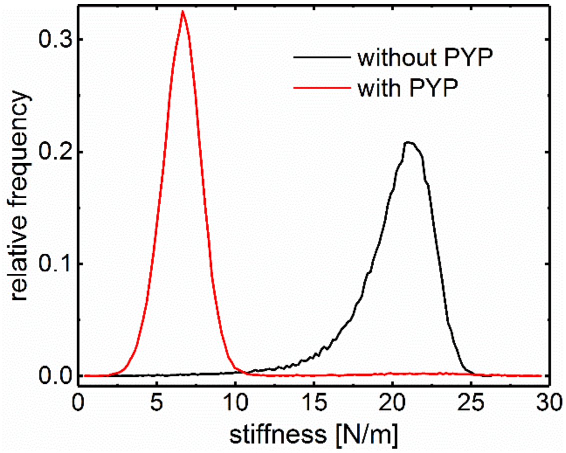

Figure S5. Histogram of stiffness values from the entire images (b) and (d) in figure S4. A lower stiffness for the PEI+(PGA+PLL)$_{6.5}$+PYP case (red curve) is consistent with the formation of an intact protein layer.

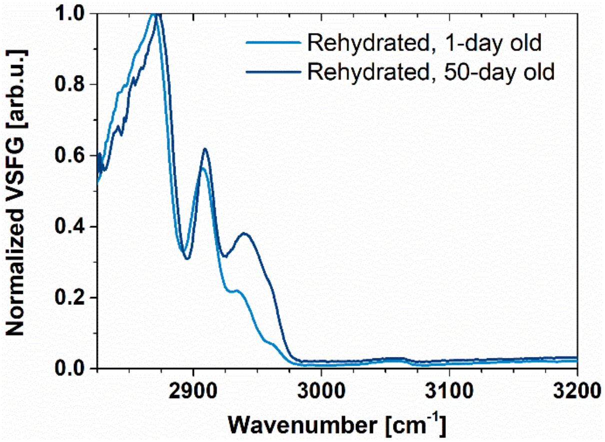

Figure S6. Structural stability of a PEI+(PGA+PLL)$_{6.5}$+PYP film over time, i.e., 1 day and 50 days after film assembly. The film was rehydrated for at least two hours before the measurements.
